# Machine learning analysis of extreme events in optical fibre modulation instability

**DOI:** 10.1038/s41467-018-07355-y

**Published:** 2018-11-22

**Authors:** Mikko Närhi, Lauri Salmela, Juha Toivonen, Cyril Billet, John M. Dudley, Goëry Genty

**Affiliations:** 1 Tampere University of Technology, Laboratory of Photonics, FI-33101 Tampere, Finland; 2 Institut FEMTO-ST, Université Bourgogne Franche-Comté, CNRS UMR 6174, 25000 Besançon, France

## Abstract

A central research area in nonlinear science is the study of instabilities that drive extreme events. Unfortunately, techniques for measuring such phenomena often provide only partial characterisation. For example, real-time studies of instabilities in nonlinear optics frequently use only spectral data, limiting knowledge of associated temporal properties. Here, we show how machine learning can overcome this restriction to study time-domain properties of optical fibre modulation instability based only on spectral intensity measurements. Specifically, a supervised neural network is trained to correlate the spectral and temporal properties of modulation instability using simulations, and then applied to analyse high dynamic range experimental spectra to yield the probability distribution for the highest temporal peaks in the instability field. We also use unsupervised learning to classify noisy modulation instability spectra into subsets associated with distinct temporal dynamic structures. These results open novel perspectives in all systems exhibiting instability where direct time-domain observations are difficult.

## Abstract (web summary)

Real-time characterisation of nonlinear processes in the time domain is challenging. Here, Närhi et al. show that machine learning techniques can help overcome this limitation and use them to infer time-domain properties of optical fibre modulation instability from spectral intensity measurements.

## Introduction

A characteristic feature of many nonlinear dispersive systems is the process known as modulation instability (MI), whereby noise on an input signal can be exponentially amplified to create localised structures of high intensity^1,2^. There has been significant interest in studies of MI in nonlinear Schrödinger equation (NLSE) systems, with many experiments reported in fibre optics, hydrodynamics and other fields^3^.

When seeded by noise, the localised structures emerging from MI show complex dynamics and random statistics, and it has even been suggested that MI may be linked to the development of extreme events or rogue waves^4–6^. Such studies have been of particular interest in nonlinear fibre optics because recent developments in real-time measurement techniques^7,8^ have allowed the emergent dynamics to be characterised experimentally in both the temporal and spectral domains. Specifically, in the temporal domain, although optical MI typically occurs on timescales that preclude direct electronic measurement, time-lens magnification has been used to characterise picosecond random breathers and solitons^9,10^. In the spectral domain, the dispersive Fourier transform (DFT) has permitted real-time characterisation of a range of instabilities in both optical fibres and laser cavities^11–17^.

These new real-time measurement techniques have essentially revolutionised the study of ultrafast instabilities in nonlinear fibre optics^18–20^, but they nonetheless remain limited in several important respects. For example, time-lens magnification is experimentally complex, typically involving a nonlinear wavelength conversion process which constrains the measurement bandwidth and power. As a result, there are relatively few experiments that have directly measured ultrafast (picosecond or shorter) extreme events in the time domain^9,10^. The DFT technique is experimentally simpler because it involves only propagation in dispersive fibre, but is typically associated with a relatively low dynamic range of 20–25 dB^21^. This is a significant limitation to the detailed study of extreme events in MI which are associated with extension in the spectral wings below the −40 dB level^22,23^.

In this paper, we describe the development of a new high dynamic range real-time spectrometer that allows the measurement and analysis of unstable MI spectra with an experimental dynamic range approaching 60 dB. Although our measurements are performed in the spectral domain, the application of machine learning to our data allows us to nonetheless compute corresponding statistics for the maximum intensity of the localised temporal peaks in the MI field, peaks that are preferentially associated with rogue wave events. Our approach employs a supervised learning algorithm, first using data from numerical simulations to train a machine learning model (based on a neural network) to correlate the complex spectral and temporal properties of noise-driven MI. We then apply the trained network to analyse high dynamic range experimental measurements of MI spectra in an optical fibre system, and from these data we determine a probability density function for the associated peak shot-to-shot temporal intensity maxima. The temporal probability density function obtained from the experimental spectra is found to be in excellent agreement with numerical modelling, including in the distribution tails that contain the high intensity extreme events. In addition to supervised learning analysis, we show how unsupervised learning can classify noisy MI spectra into subsets associated with distinct temporal dynamic structures. In particular, we show using simulations that machine learning can identify spectral clusters physically associated with different localised breather and rogue wave solutions of the NLSE^6^. Aside from the direct relevance of our results to optics, our approach has a far wider impact in showing how machine learning applied to only spectral data can be successfully used to study the properties of extreme events in the time domain.

## Results

### Modulation instability and machine learning

Machine learning is an umbrella term that describes the use of statistical techniques to analyse data sets with the aim of detecting patterns and building predictive models. Machine learning has been widely used in areas such as control systems, speech processing, neuroscience and computer vision^24^, and has very recently been applied to predicting the behaviour of chaotic systems^25,26^. Applications of machine learning in the field of photonics is also relatively recent, but a number of studies have been reported in laser optimisation^27,28^, ultrashort pulse measurements^29^, label-free cell classification^30^, imaging^31–33^ and coherent communications^34^. In our case, we aim to apply the techniques of machine learning to the study of chaotic nonlinear dynamics in optics, with the particular aim of studying the statistics of the maximum intensity of temporal peaks in noise-seeded modulation instability using only spectral measurements.

Machine learning algorithms are usually described in terms of two classes: supervised and unsupervised learning^24^. With supervised learning, prior knowledge of how the input and output of a system are related is used to build a function or model that describes the system response. With unsupervised learning on the other hand, the analysis is more exploratory, and an algorithm will search for inherent patterns and structures in a data set without using any a priori knowledge about the data or system. Here, we have applied both unsupervised and supervised learning to analyse the shot-to-shot spectral fluctuations in noise-seeded MI, and we find that they provide complementary and important insights.

We begin by presenting results applying supervised learning to analyse spectral data from noise-seeded MI. This first involves a training step where a set of MI data with known spectral and temporal characteristics is fed into a neural network to determine a transfer function capable of correlating desired input and output properties. To this end, we use stochastic numerical simulations of a generalised NLSE model to generate a large ensemble of training data (both temporal and spectral) associated with a chaotic MI field. The simulations are parameterised to model our experiments (described below) where MI develops from picosecond pulses injected into the anomalous dispersion regime of a nonlinear optical fibre. The simulations consider input pulses of 3 ps duration (full width at half maximum (FWHM)) and 175 W peak power evolving over a propagation distance of 0.68 m. The MI is seeded from a broadband quantum-limited one photon per mode spectral noise background^35^. See Methods for further details. It is important to note here that the NLSE simulation model used has been previously shown to provide a very accurate quantitative description of the statistical and noise properties of MI, supercontinuum generation and optical turbulence^9,10,13,36,37^. This is essential for its use in training the network to subsequently process experimental data. We also note that the use of numerical data to train a network prior to analysing experimental results has previously been used in ultrashort pulse measurement applications^29^.

**Fig. 1:**
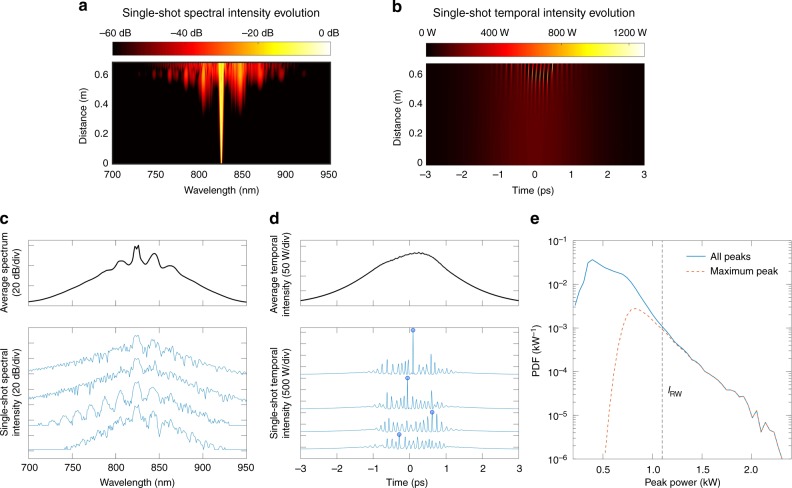
Simulated MI dynamics from picosecond pulse propagation in optical fibre. **a**, **b** Results are shown from a single simulation illustrating spectral and temporal evolution over 0.68 m of propagation. **c** Spectral data at the fibre output from multiple simulations: the top figure shows the average spectrum over 50,000 realisations; the bottom figure shows the spectral output from four realisations to illustrate the complexity of the spectra and the shot-to-shot variations. **d** Temporal data at the fibre output from multiple simulations: the top figure shows the average temporal intensity over 50,000 realisations; the bottom figure shows the temporal intensity from the four realisations corresponding to **c** to illustrate the strong temporal modulation observed. The peak intensity (maximum instantaneous peak power) in each case (shown by a circle) is the parameter we are aiming to predict from the corresponding single-shot spectra. **e** Calculated probability density function of temporal intensity peaks from simulation data. The solid line shows results from all peaks (over a 1.5 ps window), while the dashed line shows only the distribution of the maxima temporal intensity peaks. *I*_RW_ marks the rogue wave intensity threshold, defined as twice the mean intensity of the highest third of all the temporal peaks

Typical results from a single simulation showing the spectral and temporal evolution with distance are plotted in Fig. 1. We see the growth of distinct MI sidebands in the spectral domain (Fig. 1a) associated with the development of a strong modulation and emergence of localised breathers on top of the pulse envelope (Fig. 1b). In the picosecond regime, MI dynamics are highly sensitive to input noise, and for identical initial pulses but with a different random noise background, the spectral and temporal evolution can vary dramatically. This is shown explicitly in Fig. 1c, d where we plot four output spectral and temporal intensity profiles for different random noise seeds (see Methods), as well as the corresponding average profiles calculated over a larger number of 50,000 realisations. The single-shot profiles clearly show complex structure and vary dramatically from shot to shot, but of course these instability characteristics are not seen when the spectra and temporal profiles are averaged. It is for this reason that real-time measurement techniques have proven so valuable in understanding the nonlinear dynamics of MI.

These simulation results allow us to gain insight into the statistics associated with the shot-to-shot variations of MI^38^. To this end, the solid line in Fig. 1e plots the probability density function (PDF) of the intensity of the localised MI peaks across the pulse envelope. This PDF is calculated from the ~10^6^ temporal peaks identified from analysing the structure on the temporal envelopes obtained from the ensemble of 50,000 realisations. This probability distribution shows typical characteristics of MI with an extended tail, and the dashed vertical line shown in the tail region indicates the rogue wave threshold intensity *I*_RW_ defined as *I*_RW_ = 2*I*_1/3_ where *I*_1/3_ is the mean intensity of the highest third of intensity peaks.

In the context of relating MI dynamics to the appearance of extreme events and rogue waves, our aim is to determine the intensity of the maximum peak occurring in a given (single-shot) temporal profile (i.e., the points indicated by circles in Fig. 1d) from only the corresponding spectral intensity profile. Note that the associated PDF of these maximum intensity peaks from the simulation data is shown as the red dashed line in Fig. 1e. It is clear that by focussing on the maximum intensity peak from each realisation, we preferentially select out those events which have a greater probability to be classified as rogue wave events from the full distribution.

However, determining the magnitude of these temporal peaks from only spectral intensity profiles (without spectral phase) is a difficult problem because of the complexity of the noisy MI spectral characteristics. Moreover from an experimental point of view, the highest temporal peaks are associated with broad exponentially decaying spectral wings extending over many 10s of dB dynamic range, and determining the spectral bandwidth is not straightforward when dealing with noisy spectra consisting of multiple breathers with random amplitude and phase. As we will see, however, when combined with our novel experimental technique for real-time high dynamic range spectral measurements, machine learning provides a robust and convenient solution that solves this problem.

**Fig. 2:**
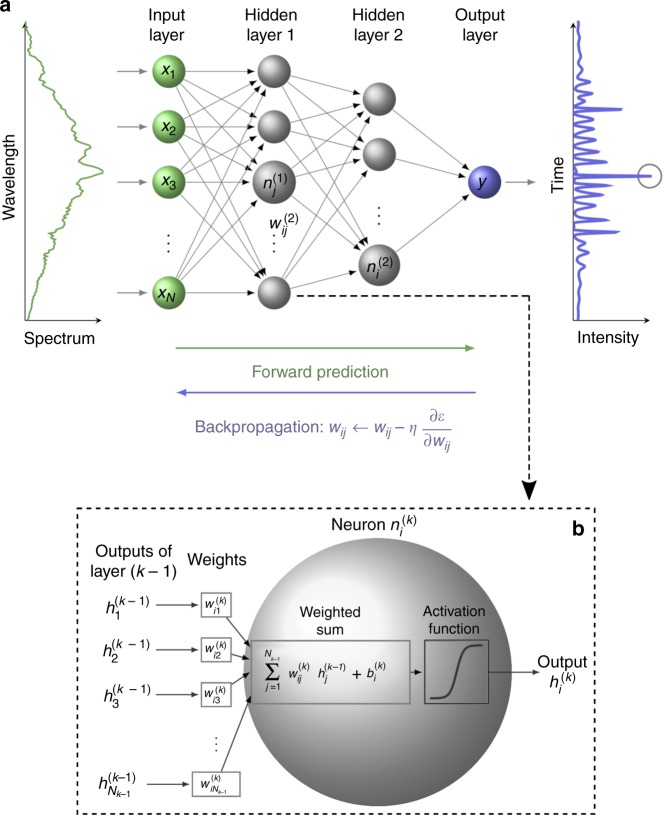
Schematic of the neural network model used to correlate spectral and temporal characteristics of MI. **a** A spectral intensity vector **X**_*n*_ = [*x*_1_, *x*_2_...*x*_*N*_] is input to a feed-forward neural network consisting of two hidden layers and a single output node that yields output *Y*_*n*_ corresponding to the maximum intensity in the time-domain intensity profile (the circled peak). The weights $w_{ij}^{(k)}$ of the network nodes correspond to the arrows connecting the node $n_i^{(k)}$ in layer *k* to node $n_j^{(k - 1)}$ in layer *k* − 1 and they are adjusted during back propagation towards the negative gradient of the error function *ε* with step size *η*. **b** Operation of a single node $n_i^{(k)}$ in layer *k* connected to *N*_(*k* − 1)_ different nodes in layer *k* − 1. See Methods for details

The specific approach we use is supervised machine learning based on a feed-forward neural network to relate the input (spectral intensity profile) and output (temporal intensity maximum) obtained from simulations. This is illustrated in Fig. 2 (see Methods for further details). In particular, the spectral intensity from a single simulation realisation with index *n* is written as a vector input **X**_*n*_ = [*x*_1_, *x*_2_...*x*_*N*_] where *x*_*i*_ is the spectral intensity at wavelength *λ*_*i*_ and mapped via a neural network to a scalar output *Y*_*n*_ corresponding to the maximum intensity of the associated temporal profile. The objective here is to use the training data to determine the weights and biases of the constituent nodes (neurons) that allow the network to perform as a transfer function to link **X**_*n*_ and *Y*_*n*_. In our case, the neural network was trained using data from an ensemble of 30,000 simulations. The typical dynamic range of the simulation spectra (between the pump and the MI wings) was ~60 dB, and anticipating the use of this network on experimental data, the spectra were also pre-processed to account for experimental conditions such as wavelength response and system resolution (see Methods).

**Fig. 3:**
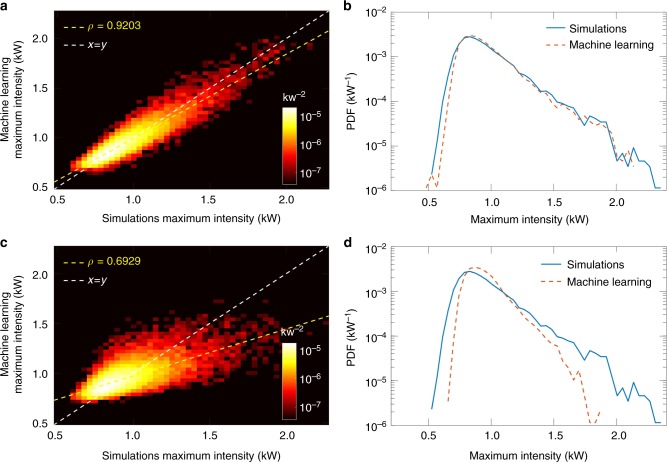
Supervised learning analysis of 50,000 simulated MI spectra. **a**, **c** A comparison is shown of the “predicted” maximum intensity (instantaneous peak power) from the machine learning algorithm with the exact “target” value from the simulated time-domain data. The top and bottom panels correspond to truncating the input spectra data at 60 dB (except on the long wavelength side, see main text) and 25 dB dynamic range (typical for standard fibre-DFT), respectively. The results in **a** and **c** are shown as a false colour representation of a histogram which shows in logarithmic scale the normalised density of points grouped into bins of constant area. The dashed white line marks the 1-to-1 correspondence between the maximum intensity (instantaneous peak power) predicted by the machine learning algorithm with the exact value from the simulated time-domain data. The value *ρ* in the legend is Pearson's correlation coefficient. **b**, **d** Probability density function (PDF) of the maximum temporal intensities predicted by the machine learning algorithm (red line) compared with the PDF calculated from the simulated time-domain data (blue line). An ensemble of 20,000 simulated single-shot spectra (distinct from the training data) was used for the comparison

After training, the model was tested on 20,000 simulations from a distinct ensemble of data not used in the training step. The aim here is to test how well the transfer function obtained from training is able to estimate the maximum temporal intensity from a new simulated single-shot spectrum, by comparing the value obtained from the machine learning algorithm with the known value from the time-domain simulation data. The results of this test are shown in Fig. 3. Here Fig. 3a shows a false colour density plot of the “predicted” maximum temporal intensity against the “target” value extracted from the simulation temporal data for the 20,000 test realisations. In order to highlight the grouping of data points, the density plot uses a histogram representation where the data points are grouped into bins of constant area. The colour scale shown corresponds to the normalised density of points in a particular bin. Note also the log scale for better visualisation. We see clear grouping around the expected *x* = *y* linear relationship (white dashed line), with very strong correlation (Pearson's correlation coefficient *ρ* = 0.92).

It is especially important to test the ability of the machine learning model to reproduce the statistics of the MI temporal peaks, and this comparison is shown Fig. 3b. The blue line shows the known PDF of the maximum intensity peaks from the simulation test ensemble, whereas the corresponding PDF determined by the machine learning algorithm is shown as the red dashed line. It is clear that the algorithm performs impressively in reproducing the shape of the probability distribution, especially the slope of the distribution tail (over nearly three orders of magnitude) as it extends to the regime of higher intensity extreme events. In this context, we note that although this agreement might be expected because the training and simulation data are generated from the same numerical model (and indeed possess indistinguishable average spectra), the purpose of the testing step is to evaluate how well the neural network has been configured during the training to yield an accurate mapping from input to output when correlating spectral and temporal properties.

As a further test during this evaluation phase, we examined whether lower-dynamic range spectral measurements (e.g., from conventional fibre-based DFT) could also be suitable for such machine learning analysis. To this end, we repeated the neural network training but truncated the dynamic range of the spectra applying a reduced dynamic range limit of 25 dB which is typical for real-time fibre-DFT systems. We plot the corresponding three-dimensional histogram results obtained when applying the network algorithm to the 20,000 test data in Fig. 3c. From Fig. 3c it is clear that there is greatly reduced visual grouping around the one-to-one relationship (white dashed line) and indeed the Pearson's correlation coefficient here is only *ρ* = 0.69. Moreover, the predicted PDF shown in Fig. 3d fails to reproduce the slope of the tail, emphasising the importance of the high dynamic range in capturing extreme events. Note that we performed similar tests over a wider range of parameters, and found that machine learning was only able to construct a reliable model when the spectral data possessed a dynamic range exceeding 50 dB. It is of course also important in this regard to ensure that the training and experimental conditions are as close as possible, and the network performance will be reduced if this is not the case. For example, applying the network algorithm as trained above to an ensemble of test data generated with a ±20% difference in input pulse peak power yields a PDF with an overall shape similar to that expected from simulations, but the algorithm in this case does not reproduce the slope of the tail of the PDF corresponding to the highest maximum intensities.

In addition to the supervised learning approach described above, we also applied unsupervised learning to analyse the MI process. The motivation here is to automatically classify unstable MI spectra into subsets associated with different classes of localised breather structures possessing particular analytic solutions^38^. Because of the very complex nature of these spectra with much low-amplitude fine structure (e.g., see Fig. 1c), this is a challenging objective, but as we show below, machine learning succeeds in performing such classification.

The approach used was to apply a clustering algorithm to partition a large ensemble of simulated spectra into distinct clusters whose structure exhibits similarity based on a distance metric relative to the cluster centroids (see also Methods)^39^. Note that such an algorithm does not classify the spectra using a single measure such as bandwidth or amplitude, but rather identifies clusters based on the structure of the spectra over their full bandwidth, making it sensitive to complex features such as sidebands, fine structure and the slope of low-amplitude wings.

In our case, we ran a *k*-means clustering algorithm on an ensemble of 50,000 simulations and found that a partition number *k* in the range 5–30 yielded similar results. In particular, independent of the number of clusters used, the results showed that the spectra in the cluster with the largest population were associated with temporal profiles whose maximum intensity was close to the maximum of the probability distribution (Fig. 1e). In addition, the mean bandwidth of the spectra in this cluster was closest to that calculated from all the spectra in the ensemble. Significantly, this result allows us to expect on physical grounds that the temporal profiles of the highest intensity peaks corresponding to the spectra in this cluster would be well fitted by the Akhmediev breather solution to the NLSE at the point of the maximum MI gain, as this is the breather solution which dominates the dynamics^38^. Similarly, we were able to confirm that the cluster with the smallest population grouped together spectra whose associated temporal profiles had maximum intensities in the tail of the probability distribution, and the spectra in this cluster had the largest calculated mean bandwidth. Again on physical grounds, we can then expect these spectra to be associated with temporal peaks corresponding to the strongly localised Peregrine soliton solution of the NLSE, and perhaps even higher-amplitude profiles associated with breather collisions^9^.

**Fig. 4:**
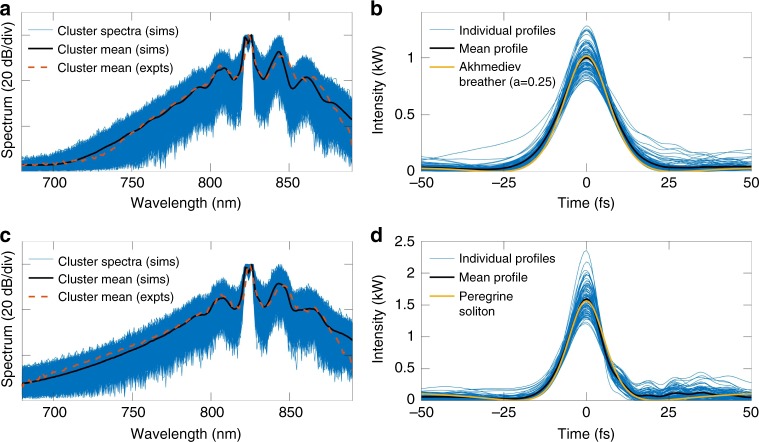
Unsupervised learning analysis of 50,000 simulated MI spectra. **a** Superposed blue curves show the cluster of spectra whose mean is closest to the mean spectrum calculated from the full ensemble. The average of this cluster is the black line. The corresponding temporal data is shown in **b** where the superposed blue curves show the profiles of the highest intensity peaks in the time-domain field associated with the spectra in **a**. The average temporal profile is shown in black and compared with the analytic Akhmediev breather profile (yellow) calculated at the maximum MI gain. **c**, **d** Shown are corresponding results for the cluster of spectra exhibiting the largest mean bandwidth. The yellow curve in the time-domain data plotted in **d** is the analytic Peregrine soliton. The red dashed lines in **a** and **c** show the average spectra calculated from clustered experimental data under the same conditions as simulations

To show this explicitly, Fig. 4 presents results obtained using 9 clusters. Firstly, we consider the results in Fig. 4a, b which show spectral and temporal profiles associated with the cluster of spectra closest to the mean of the spectrum across the whole ensemble. This cluster includes 8402 elements. The superposed blue curves in Fig. 4a show individual spectra from this cluster, while the black curve shows their calculated mean. The individual temporal profiles of the highest intensity peaks in the corresponding time-domain fields are shown as the superposed blue curves in Fig. 4b and the black line plots the mean of these temporal peaks. The yellow line plots the analytic Akhmediev breather at maximum MI gain (see Methods), and we note the excellent agreement between the cluster mean and the analytic breather solution.

The results in Fig. 4c, d show spectral and temporal profiles associated with the cluster of spectra with largest mean bandwidth and which includes 2153 elements. Again, the superposed blue curves in Fig. 4c show individual spectra while the black curve shows their calculated mean. The individual temporal profiles of the highest intensity peaks in the corresponding time-domain fields are shown as the blue curves in Fig. 4d and the black line plots the mean of these temporal peaks. The yellow line here plots the analytic Peregrine soliton (see Methods), and we note again the excellent agreement between the cluster mean and the analytic solution.

### Experimental setup and results

Our experimental setup was designed to measure a large ensemble of high dynamic range spectra from noise-seeded MI, suitable for analysis using machine learning as described above. To this end, we first generated a noisy MI field by injecting 3 ps duration (FWHM) pulses of 175 W peak power into 0.68 m of photonic crystal fibre (PCF) with zero-dispersion wavelength around 750 nm. At the pump wavelength of 825 nm, the fibre exhibits strong anomalous dispersion such that clear characteristics of MI are observed. The pump source used was an 80 MHz mode-locked Ti:Sapphire laser. Note that the simulations described above used identical parameters to these experiments.

**Fig. 5:**
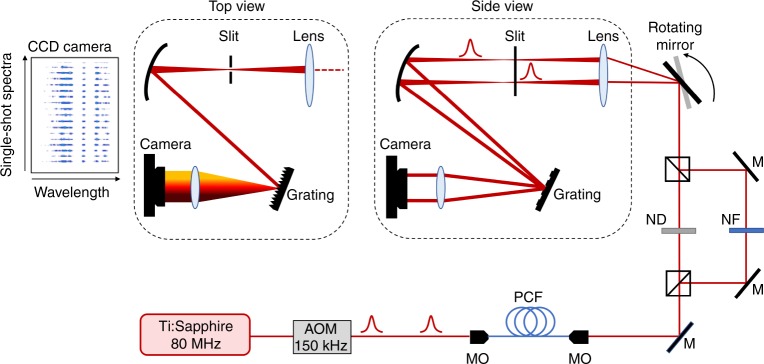
Experimental setup. Ti:Sapphire Titanium-Sapphire mode-locked laser, AOM acousto-optic modulator, MO microscope objective, PCF photonic crystal fibre, M mirror, ND neutral density filter, NF notch filter, CCD charge coupled device. Side views and top views of the grating imaging setup are shown

To characterise the shot-to-shot spectra with high dynamic range, we developed a novel real-time spectrometer setup as shown in Fig. 5. We first reduce the MI signal repetition rate to 150 kHz (using an acousto-optic modulator placed after the Ti:Sapphire laser) and use a rapidly rotating mirror to scan sequential spectra onto different vertical positions of the entrance slit of a 1.1 nm resolution Czerny–Turner spectrograph. Most importantly, this approach is combined with spectral windowing and differential attenuation to capture the central region and the lower amplitude wings of the individual spectra separately, such that the two distinct spectral regions are recorded with the full available dynamic range of the detector. Post-processing is then used to recombine the two windowed components, yielding a dynamic range approaching 60 dB, a near four-order magnitude improvement compared to conventional fibre-DFT. See Methods for further details.

**Fig. 6:**
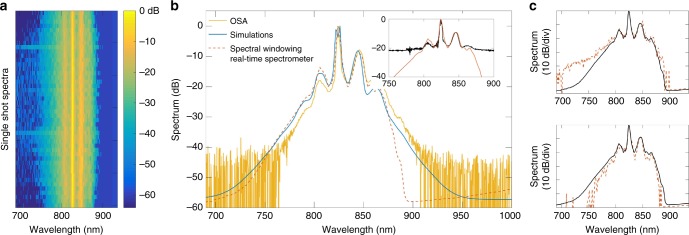
Experimentally recorded single-shot MI spectra with high dynamic range. **a** Series of 60 recorded spectra stacked along the vertical direction to illustrate the shot-to-shot fluctuations seen with MI. **b** Average spectrum measured with our high dynamic range real-time technique (dashed red), average spectrum measured with an optical spectrum analyser (yellow), and simulated average spectrum (blue). The inset shows the comparison between our 60 dB dynamic range spectral measurement (dashed red) and that using a conventional fibre-DFT with only ~22 dB dynamic range (black). **c** Two selected single-shot spectra from experiments (dashed red) compared with the average experimental spectrum (solid black)

Figure 6 shows experimental results where this technique was used to measure an ensemble of 3000 MI spectra. Firstly, Fig. 6a shows a sequence of 60 consecutively recorded spectra to illustrate how the real-time measurements capture the large shot-to-shot fluctuations expected from MI in the picosecond regime^9,12,35^. It is especially significant that the high dynamic range clearly reveals variations in the structure of the spectral wings below the −40 dB level. As a check on the fidelity of these measurements, we computed the mean of the 3000 real-time spectra (dashed red line in Fig. 6b) to compare with an independent measurement (solid yellow line) using an integrating optical spectrum analyser (OSA). We see very good agreement between the OSA measurement and the average of the real-time measurements for the central region, the MI sidebands and the slope of the wings on the short wavelength edge. Note that the discrepancy observed for wavelengths beyond 875 nm in the wings when compared to the OSA is due to reduced throughput efficiency of the system (i.e., grating and camera response).

To further highlight the advantage of the windowed-real-time technique developed here, the inset to Fig. 6b compares the average from our high dynamic range measurements with the results of additional experiments where we used a standard fibre-DFT setup with dynamic range of ~22 dB (see Methods for details). The near four orders of magnitude improvement using spectral windowing is very apparent from this comparison. To show explicitly how this enhanced dynamic range reveals shot-to-shot differences in the spectral wings, Fig. 6c compares the structure of two measured single-shot spectra (dashed red line) with the average computed over the 3000 measured spectra (solid black line). We also plot on this figure (blue solid line) the mean spectrum calculated from the full ensemble of 50,000 numerical simulations of our experiments as described above. In this context we note that for the supervised machine learning training step, the simulated spectra were multiplied by a spectral response function to match the experimental fall off above 875 nm. This ensures that the model obtained from training using simulations can be applied to experimental results.

**Fig. 7:**
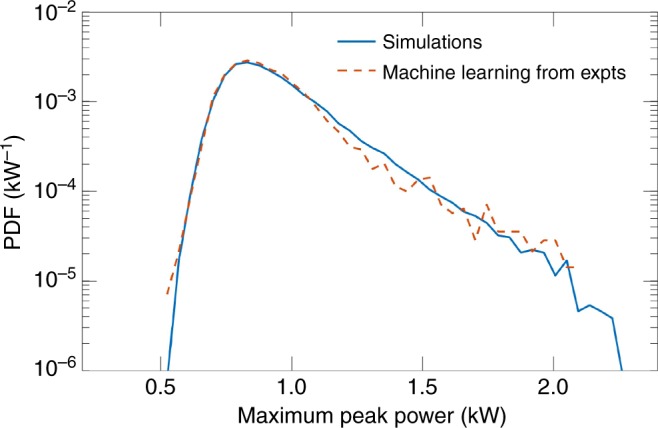
Probability density function (PDF) of the maximum intensity of the temporal intensity profiles predicted by the machine learning algorithm from an ensemble of 3000 single-shot experimental spectra (dashed red line) compared with the PDF calculated from the simulated time-domain data (solid blue line)

Results applying the trained supervised learning model to the experimental data are shown in Fig. 7. Here, we aim in particular to determine from the measured single-shot spectra the associated maximum temporal intensity. Figure 7 plots the PDF obtained from the machine learning analysis of the experimental data (dashed red) compared with that from numerical simulations (solid blue). We see very good agreement between the experimental and simulation probability density functions, especially in the slope of the distribution tail for the highest intensity extreme events. These results show very clearly that even though the only available experimental data are that of the spectral intensity, we can nonetheless extract significant physical information about the corresponding temporal behaviour, and in particular reproduce the long tail of the statistics associated with the emergence of localised breather and rogue wave structures.

We also ran the unsupervised learning clustering algorithm on the ensemble of 3000 experimental spectra to cluster the spectra into 9 partitions as in our analysis of the simulation data. These results are shown in Fig. 4. Specifically, the dashed red line in Fig. 4a plots the mean of the partition whose bandwidth is closest to that of the mean of the full ensemble, while the dashed red line in Fig. 4b plots the mean of the cluster with largest spectral bandwidth. We see how the spectral clusters identified from the experimental data closely match those from the numerical simulations.

## Discussion

There are several major conclusions to be drawn from these results. Firstly, for the modulation instability system studied here, we have shown that real-time measurements of only the spectral intensity can be combined with supervised machine learning to yield quantitative information about temporal characteristics using training based on accurate numerical simulations. In particular, by relating spectral characteristics to the maximal intensity of the corresponding time-domain peaks, we can extract a probability distribution that preferentially selects out events which satisfy rogue wave criteria. Since this allows the presence of deleterious high-power temporal spikes to be captured even though only optical spectra are being measured, this is of potential practical significance in imaging and spectroscopy experiments using supercontinuum sources seeded by an initial regime of modulation instability. Secondly, our simulation results showing the ability of unsupervised machine learning to cluster a large ensemble of modulation instability spectra into different classes associated with specific dynamical structures is another important aspect of our work. A further significant element of our results concerns the experimental technique used to capture the shot-to-shot instability spectra with high dynamic range. By windowing the complex modulation instability spectra and using differential attenuation, it has been possible to measure spectra in real time and with nearly 60 dB dynamic range. Being able to characterise spectra with such a large dynamic range is an essential component in successfully applying machine learning to our data. This approach is experimentally straightforward and can be implemented at all wavelengths where suitable spectrometers are available. In this context we note that it is a long standing problem in ultrafast optics to relate temporal and spectral information when only the intensity properties are known; the underlying fields also contain phase components, and it is generally extremely difficult to correlate temporal and spectral properties without this phase information. The possibility to infer time-domain properties in optics only from real-time spectral measurements that are easier to implement experimentally is significant not only from the point of view of studying the particular process of modulation instability as we do here, but also more generally in the field of ultrafast optics. We also note in this context that the use of accurate simulations to train a neural network subsequently applied to experimental data opens up many possibilities for the applications of machine learning in optics. Indeed, the use of simulation-based training applied to real-world data (”sim-real transfer”) is a burgeoning field of machine learning, and with the wide availability of realistic numerical models for many propagation scenarios in both linear and nonlinear optics, we anticipate many future applications in the analysis of optical systems.

Finally, although demonstrated here in an optical context, the principle of using machine learning to study temporal properties of a nonlinear system based only on spectral intensity measurements would be expected to apply to many physical systems exhibiting chaos and instability where direct time-domain observations are precluded.

## Methods

### Numerical modelling

Our numerical modelling is based on the well-known generalised NLSE model describing the evolution of a field envelope in an optical fibre^35^. This model has been previously shown to provide a very accurate quantitative description of the statistical and noise properties of MI and supercontinuum generation^13,36^. Here, we model the propagation of 3 ps (FHWM) *P*_0_ = 175 W peak power hyperbolic-secant pulses in the anomalous dispersion regime of a 68 cm-long PCF (NKT Photonics NL-PM-750) with Taylor-series expansion dispersion coefficients at 825 nm: *β*_2_ = −1.03 × 10^−26^ s^2^ m^−1^, *β*_3_ = 4.74 × 10^−41^ s^3^ m^−1^, *β*_4_ = 2.35 × 10^−56^ s^4^ m^−1^, *β*_5_ = −1.17 × 10^−70^ s^5^ m^−1^ and *β*_6_ = −9.07 × 10^−85^ s^6^ m^−1^. The nonlinear coefficient *γ* = 0.1 W^−1^ m^−1^. For completeness, we also include the Raman and shock terms in the model, but for our parameter regime, these had minor influence on the dynamics (although the Raman effect does lead to the observed MI sideband asymmetry.)

Simulations used 4096 grid points with a temporal window of 12 ps corresponding to an 83 GHz spectral resolution. Noise was included in the frequency domain via a one photon per mode spectral background with random phase to seed the growth of MI sidebands outside the bandwidth of the pump pulse. This random phase background in the initial conditions varies between different simulation realisations. We generated an ensemble of 50,000 numerical simulations corresponding to different input noise seeds with 30,000 used for training and 20,000 for testing of the neural network. To account for experimental shot-to-shot fluctuations introduced by using an acousto-optic modulator (AOM) to operate at reduced repetition rate, the simulations also included an additional ±5% random variation of input peak power between different realisations in the ensemble. Because the MI modulation frequency at maximum gain is given by *Ω* = (2*γP*_0_/|*β*_2_|)^1/2^, this leads to a very small variation in the temporal modulation frequency on the pulse envelope for each realisation in the ensemble. However, the intrinsic randomness of the temporal structure from shot to shot is determined by the initial background spectral noise which is amplified nonlinearly by the MI process. Indeed, without the one photon per mode broadband noise background, the simulations do not show the growth of MI at all.

### Analytic breather and Peregrine soliton solutions for MI

We give here the analytic form of the Akhmediev breather and Peregrine soliton solutions of MI that are plotted for comparison with the clustering results in Fig. 4. Akhmediev breathers form a one-parameter class of localised solutions of the NLSE, and at their point of maximum localisation (maximum amplitude and minimum temporal width), their analytic form in dimensional units is given by:

1 $$A_{{\mathrm{AB}}}(t) = \sqrt {P_0} {\kern 1pt} {\kern 1pt} \frac{{(1 - 4a) + \sqrt {2a} {\mathrm{cos}}(\omega _mt)}}{{\sqrt {2a} {\mathrm{cos}}(\omega _mt) - 1}},$$

where parameters 0 < *a* < 0.5 and *ω*_*m*_ are related by $2a = 1 - \omega _m^2|\beta _2|/(4\gamma P_0)$.

The solutions plotted in Fig. 4 are the Akhmediev breather solution corresponding to the frequency of maximum MI gain where *a* = 0.25 in Eq. (1), and the Peregrine soliton solution which is obtained in the asymptotic limit *a* = 0.5. In this latter case, the Peregrine soliton takes the rational form:

2 $$A_{{\mathrm{PS}}}(t) = \sqrt {P_0} \left[ {1 - 4/\left( {1 + 4\gamma P_0t^2/|\beta _2|} \right)} \right].$$

Note that the parameters used for the analytic solutions in Fig. 4 are the experimental values given in the preceding section. Finally we remark that the simulation and analytical intensity profiles are normalized according to the common convention in nonlinear fibre optics to show instantaneous power^35^.

### Machine learning

Machine learning describes the use of computational and statistical techniques to analyse data sets with the aims of classifying data and building models^24^. Machine learning algorithms are usually described in terms of two classes. A supervised learning algorithm uses classification and regression techniques to train a model from a known set of input and output data such that the model can be used to map new inputs to new outputs. In contrast, unsupervised learning is used to find patterns or intrinsic structures in data sets without any a priori knowledge of the system or data properties. We now present further details of how these techniques were used here.

### Supervised learning

The goal of supervised learning is to use a set of known ”training” data to determine a function or model that will map an input to an output. In our case the input **X** represents the intensity spectrum of a modulation instability field, while the output *Y* represents a single number—the intensity of the highest peak in the corresponding temporal intensity profile.

Our training data are obtained from an ensemble of 30,000 numerical simulations, and we denote the training data pairs as (**X**_*n*_, *Y*_*n*_) with *n* = 1…30,000. The mapping function used in supervised learning can take various forms including decision trees, regressions, neural networks or Bayesian classifiers^40,41^, and in our implementation, we used a multilayer neural network as shown in Fig. 2. Such a network consists of basic computational units (nodes or ”neurons”) organised into different layers: an input layer which accepts the input data **X**_*n*_, intermediate hidden layers that perform operations on the data and the final layer which computes the network output. Each node in a given layer accepts multiple inputs from the previous layer and these are weighted, summed and combined with an additive bias to yield a resulting single real-value which is passed to an ”activation function” to generate the node output.

In more detail, the network we used consisted of an input layer, two dense (i.e., fully connected) layers of hidden nodes with a nonlinear activation function and an output layer. The two dense hidden layers had 30 and 10 nodes, respectively. The output layer is a single linear node. The output of a generic node $h_i^{(k)}$ in layer *k* is calculated by combining the outputs $h_j^{(k - 1)}$ from the previous *k* − 1-th layer:

3 $$h_i^{(k)} = f\left( {\mathop {\sum}\limits_{j = 1}^{N_{k - 1}} w_{ij}^{(k)}h_j^{(k - 1)} + b_i^{(k)}} \right).$$

Here $w_{ij}^{(k)}$ are the weights between nodes $n_j^{(k - 1)}$ and $n_i^{(k)}$ of layers *k* − 1 and *k*, respectively and the summation is calculated over *N*_*k*−1_, the number of nodes in layer *k* − 1. The term $b_i^{(k)}$ represents the bias for each node $n_i^{(k)}$ in layer *k*, and *f*(*x*) = 2/[1 + exp( − 2*x*)] − 1 is the nonlinear (hyperbolic tangent sigmoid) activation function. For the output layer, a linear activation function was used. The node weights and biases are initially set to random values and then optimised using conjugate gradient back-propagation^42,43^ in order to minimise a cost function, defined as:

4 $$\epsilon = \frac{1}{N}\mathop {\sum}\limits_{n = 1}^N \left( {Y_n - Y_n^\prime } \right)^2.$$

where *N* is the number of samples in the training data, *Y*_*n*_ is the target value and $Y_n^\prime$ is the output of the network. The weights *w*_*ij*_ are iteratively adjusted by an amount Δ*w*_*ij*_ with learning rate *η* towards the negative gradient of *ϵ* such that ${\mathrm{\Delta }}w_{ij} = - \eta \frac{{\partial \varepsilon }}{{\partial w_{ij}}}$. This process is repeated over a number of ”epochs” (one forward pass and one backward pass of all (**X**_*n*_, *Y*_*n*_) training pairs through the network) until convergence (no change in the gradient descent with subsequent multiple iterations). At this point the network is suitably configured to perform as the desired transfer function linking **X**_*n*_ and *Y*_*n*_ pairs.

Because our goal is to apply the machine learning algorithm to real-world experimental data, the simulated MI spectra were pre-processed to account for experimental constraints, i.e., the wavelength-dependent response (which falls off above 875 nm) and the 1.1 nm resolution of the spectrometer. With this pre-processing, the input vector then consists of *N* = 121 uniformly distributed spectral intensity bins such that each MI spectrum from the simulation ensemble was discretised onto a vector **X**_*n*_ = [*x*_1_, *x*_2_...*x*_*N*_]. The neural network was trained for 300 epochs of all 30,000 training sets.

### Unsupervised learning

Clustering is the most common unsupervised learning technique used for exploratory data analysis. In our analysis, we used the *k*-means method that divides unclassified data into *k* mutually exclusive clusters by minimising the distance from the data to the cluster centroid. The algorithm begins with random initialisation of the centroid locations, and this is followed by a classification of the data into clusters based on distance to these centroids. The centroids of these clusters are then calculated, the cluster populations are updated based on these new centroid locations, and this process is repeated until the centroid positions stabilise. It is important to note here that the *k*-means algorithm does not cluster on a single metric such as, e.g., the spectral bandwidth or amplitude, but rather identifies the clusters of different spectra based on the structure and shape of the spectra over their full bandwidth. Indeed, it is the ability of the clustering algorithm to detect patterns in the shot-to-shot spectral structure that demonstrates its utility for this purpose.

Using this method, the 50,000 simulated spectra were classified using different number of clusters (*k* varying from 5 to 30) to ensure that any conclusions drawn would be independent of the number of clusters used. For the spectra grouped in each cluster, we calculated the corresponding time-domain intensity profile locally around the intensity maximum and examine these for all clusters. Independent of the number of clusters used, the cluster containing the largest number of spectra yielded local temporal profiles with maximum intensity close to the maximum of the probability distribution (Figs. 1e and 7). On the other hand, clusters corresponding to lower or higher intensities than the distribution maximum contained fewer spectra so that the cluster sizes essentially follow the probability distribution. In the distribution tail at the highest intensities, we found only one cluster would be identified from the algorithm, and this cluster contained the smallest number of spectra. Results in Fig. 4 illustrate the classification results using *k* = 9 for the clusters with the largest and smallest number of spectra produced by the algorithm both for the 50,000 simulated spectra and the 3000 experimental spectra. For completeness here, we give the number of elements in each cluster generated from the simulated data. Specifically for the 50,000 simulations, the cluster sizes ordered from largest to smallest population were: 8402, 6948, 6327, 5853, 5596, 5074, 4973, 4674 and 2153. Note that the results in Fig. 4 correspond to the clusters with the largest and smallest populations. For the 3000 experimental spectra, the cluster populations were found to be: 528, 526, 471, 430, 429, 317, 280, 210 and 49. Note that the algorithm does not return the clusters with any sorting order. Any physical interpretation of the clusters identified must be performed independently of the algorithm itself as we have done above by associating the spectra in particular clusters with their associated temporal properties.

### High dynamic range real-time spectrometer

Single-shot MI spectra were measured in real time at the fibre output using a rapidly rotating mirror mounted on a galvanometer (Nutfield QS-12) with angular speed *ω* = 240 rev. per min, and focussed with a lens of focal length *f* = 150 mm at the entrance slit of a Czerny–Turner spectrograph. The spectrograph used a grating with 300 lines per mm and 500 nm blaze (ThorLabs GR25-0305) to disperse consecutive spectra onto different lines of a high-sensitivity electron-multiplying charged-coupled device (EMCCD) camera (Andor iXon 3), allowing single-shot spectral intensity measurements with a 1.1 nm resolution. With this scan rate and our setup, it was necessary to reduce the repetition rate of the laser to 150 kHz using an acousto-optic modulator, but acquisition speeds up to the MHz range would be possible either using a faster galvanometer, using a multi-pass geometry^44^ or by increasing the focal length at the spectrograph entrance slit.

The camera was cooled to −80 °C and used 5× pre-amplifier gain to decrease the noise level to a single electron level corresponding to a maximum dynamic range close to 40 dB. In order to increase the effective dynamic range of the measurement, we used a differential spectral attenuation scheme that captures the central part and wings of the MI spectra separately with the same dynamic range. In this scheme, the MI field at the fibre output is divided between two arms of unequal length corresponding to a 200 ps delay. Differential attenuation was induced in the two arms using a notch filter with a 40 dB, 20 nm rejection band centred at 825 nm (Edmund Optics) and a variable neutral density filter, respectively. Beams from the two arms are then recombined with a beamsplitter such that the central part and wings of the individual spectra are recorded with the same dynamic range and 200 ps delay by the individual lines of the EMCCD. The spectral response of the system was carefully calibrated by measuring the mean spectrum with and without the filters. The full spectra are subsequently recombined by post-processing with an effective 60 dB dynamic range, representing a more than three orders of magnitude improvement compared to a conventional fibre-based DFT approach^12,13^. Direct comparison of the average MI spectrum at the PCF output was performed with an integrating optical spectrum analyser (Ando AQ6315B).

### Conventional fibre-DFT

The conventional fibre-DFT implemented for comparison with the high dynamic range real-time method used a 100 m custom fabricated fibre (IXfibre IXF-SM series) designed to be single mode over a broad wavelength range in the near-infrared and with total dispersion *β*_2_*L* = +4030 ps^2^ at 825 nm. The input to the dispersive stretching fibre was attenuated to ensure linear propagation. The real-time spectra were recorded with a 25 GHz InGaAs photodiode (UPD-15-IR2-FC Alphalas) and 20 GHz real-time oscilloscope (DSA72004 Tektronix), leading to an effective resolution of ~0.03 nm.

## Data Availability

The data that support the plots, code modules used in data analysis and other findings of this study are available from the corresponding author upon reasonable request.
